# Understanding the Interaction between a Steel Microstructure and Hydrogen

**DOI:** 10.3390/ma11050698

**Published:** 2018-04-28

**Authors:** Tom Depover, Aurélie Laureys, Diana Pérez Escobar, Emilie Van den Eeckhout, Elien Wallaert, Kim Verbeken

**Affiliations:** Department of Materials, Textiles and Chemical Engineering, Ghent University (UGent), Technologiepark 903, B-9052 Ghent, Belgium; aurelie.laureys@ugent.be (A.L.); diana.perezescobar@gmail.com (D.P.E.); emilie.vandeneeckhout@ugent.be (E.V.d.E.); elien.wallaert@ugent.be (E.W.); kim.verbeken@ugent.be (K.V.)

**Keywords:** hydrogen embrittlement, hydrogen-induced cracking, thermal desorption spectroscopy, in situ tensile testing, permeation

## Abstract

The present work provides an overview of the work on the interaction between hydrogen (H) and the steel’s microstructure. Different techniques are used to evaluate the H-induced damage phenomena. The impact of H charging on multiphase high-strength steels, i.e., high-strength low-alloy (HSLA), transformation-induced plasticity (TRIP) and dual phase (DP) is first studied. The highest hydrogen embrittlement resistance is obtained for HSLA steel due to the presence of Ti- and Nb-based precipitates. Generic Fe-C lab-cast alloys consisting of a single phase, i.e., ferrite, bainite, pearlite or martensite, and with carbon contents of approximately 0, 0.2 and 0.4 wt %, are further considered to simplify the microstructure. Finally, the addition of carbides is investigated in lab-cast Fe-C-X alloys by adding a ternary carbide forming element to the Fe-C alloys. To understand the H/material interaction, a comparison of the available H trapping sites, the H pick-up level and the H diffusivity with the H-induced mechanical degradation or H-induced cracking is correlated with a thorough microstructural analysis.

## 1. Introduction

The limited fossil fuel resources, the concerns about nuclear power, and especially global warming issues imply energy related challenges that are triggering scientists to find ecological solutions for an environmentally friendly future. Hydrogen (H) gas is an attractive replacement for fossil fuels since it is the most abundant element on earth and its combustion only generates water. Hence, no greenhouse gasses are emitted in the atmosphere [1]. Despite these advantages, H contains a negative connotation due to multiple incidents that happened in the past indicating the potential danger of its usage [2]. Another approach to make transportation more ecological is to lower the fuel consumption of vehicles. In automotive industry, the use of high-strength steels (HSS) has, for instance, been promoted since it can both guarantee an increased safety together with weight reduction, which is required to meet the stringent CO_2_ emission guidelines. Unfortunately, these steels are prone to H-induced mechanical degradation [3,4,5], which impedes further alloy development, according to BMW [6]. Steels used in offshore industry are often protected against corrosion by cathodic protection which causes a conversion of all the active anodic sites of the metal to inactive cathodic sites. However, when cathodic overprotection occurs, atomic H absorbs into the steel and a H concentration is built up. Consequently, the material integrity degrades, and H-induced failure is promoted [7].

Johnson [8] was the first to describe the potential effect of H on the mechanical properties of iron; i.e., a temporary loss of ductility was observed. This work has inspired a lot of researchers to continue investigating this subject resulting in many reference works and international conferences [9,10,11,12,13,14,15,16,17,18]. Recently, the attention got reinforced by the development of numerous applications of high-strength steels involving H interaction, such as oil and gas pipelines, vehicles, storage tanks, offshore structures, welds, etc. [19,20,21,22]. The phenomenon of temporary ductility loss implies so called hydrogen embrittlement (HE), causing unpredictable failure. Absorbed and dissolved H diffuses through the metal, introducing extra stresses at potential crack initiation sites and/or facilitating crack propagation. HSS are very susceptible to HE because of their high stress level as well as their large number of potential fracture initiation sites [3,23]. Consequently, the potential and safe application of H still offers major challenges to the materials engineer and clearly a renewed interest in this research field occurred during the last decade in both industrial and scientific communities.

Although the detrimental H effect was already discussed in 1875 [8], no full understanding of the phenomenon governing the observed H-induced ductility loss has been achieved up to now. Several mechanisms have been proposed to describe HE, but none has been fully accepted since indications accounting for different mechanisms have been observed experimentally. The three most cited mechanisms in non-hydride forming materials are the H Enhanced DEcohesion (HEDE) [24], the H Enhanced Localized Plasticity (HELP) [25] and Adsorption-Induced Dislocation Emission (AIDE) theory [26,27]. HEDE proposes a decrease in the cohesive bond strength between the metal atoms in the presence of H causing brittle crack propagation under tensile load. HELP considers an increase in dislocation mobility due to the presence of H, resulting into highly localized plastic deformation and accelerated failure. This mechanism has attained substantial support [28,29,30]. AIDE combines both HEDE and HELP since it also involves localized plasticity, but it proposes that the localized plasticity occurs close to the surface at regions of stress concentrations, for instance at crack tips, including nanovoids in front of it.

Multiphase HSS, such as transformation-induced plasticity (TRIP), dual phase (DP) and high-strength low-alloyed (HSLA) steels are commonly used in the automotive industry and were already subject of several H related studies since they are prone to HE [31,32,33]. These HSS show, however, a multiphase microstructure, complicating the interpretation of H related data. Therefore, the interaction between H and single-phase alloys is also of interest to get an improved understanding of the damaging mechanisms [34,35,36,37]. Additionally, the presence of carbides is generally considered to be beneficial to improve the HE resistance since they are assumed to trap H efficiently removing the detrimental mobile H from the microstructure [38,39]. The size and coherency of the specific carbides were crucial in terms of H trapping ability [40,41]. However, the applied H charging conditions might affect their beneficial role, as will be evaluated further in Section 5.

This work provides an overview of our work on the interaction of H with a steel microstructure and is built up in a systematic approach. At first, the effect of H charging on HSS, i.e., TRIP, DP and HSLA, will be considered. To simplify the microstructure, generic Fe-C lab-cast alloys consisting of a single phase, i.e., ferrite, bainite, pearlite or martensite and with carbon contents of approximately 0, 0.2 and 0.4 wt %, will be studied. Finally, the addition of carbides will be investigated in Fe-C-X alloys by adding a ternary carbide forming element to the Fe-C alloys. A comparison of the H absorption level, the available trapping sites and the H diffusivity with the H-induced mechanical degradation or H-induced cracking was correlated with a detailed microstructural analysis to understand the H/material interaction.

## 2. Experimental Procedure

### 2.1. Materials Characterization

#### 2.1.1. Advanced High-Strength Steels

The investigated multiphase, advanced HSS such as TRIP, DP and HSLA, contained different constituents, such as martensite, bainite, ferrite and retained austenite. The thickness of the TRIP steel sheets was 0.7 mm, while DP and HSLA steels had a thickness of 1.1 mm. These thicknesses were reached after hot and cold rolling, followed by subsequent annealing via industrial annealing parameters required to obtain the desired microstructure. TRIP steel contained ferrite, martensite, bainite and retained austenite. The amount of retained austenite, quantified by XRD, was 9.6%. The carbon content of the retained austenite was determined to be 1.2 wt % [42]. The DP steel consisted of ferrite and 23.6% martensite [43]. In the HSLA steel, cementite (Fe_3_C) was observed in the ferrite matrix, while this steel also contained Ti- and Nb-based carbonitrides [44]. Chemical compositions are summarized in Table 1. Notched tensile samples, with stress concentration factor of 4.2, were made by spark erosion, the tensile axis being parallel to the rolling direction [45].

#### 2.1.2. Pure Iron, Generic Fe-C and Fe-C-X Alloys

Generic steels were studied to avoid the effect of complex microstructural characteristics. The lab-cast generic alloys were “pure iron”, ultra-low carbon (ULC) steel and two Fe-C-based materials containing 0.2 and 0.4% of carbon. This difference in carbon content will allow evaluating the impact of the carbon content on HE. A commercial Armco pure iron was used as a reference as well. The chemical compositions are given in Table 2. The alloys were produced in a Pfeiffer VSG100 vacuum (Pfeiffer, Asslar, Hessen, Germany) melting and casting unit, operating under an argon gas protective atmosphere. The materials were hot and cold rolled resulting in sheet material with final thicknesses ranging between 1 and 2 mm while different heat treatments were applied to induce a ferritic, bainitic, martensitic or pearlitic structure, for which the authors refer to the corresponding works [46,47,48,49]. The effect of deformation-induced defects on the H behavior in Armco pure iron and ULC steel was investigated by applying varying degrees of cold deformation and heat treatments [49,50].

Additionally, the H/carbide interaction was studied extensively. Fe-C-X grades with a stoichiometric amount of a ternary alloying element X, i.e., Ti, Cr, Mo, W or V, were processed. Each grade was incrementally cast into three alloys with increasing carbon content (cf. Table 3). The carbon increase allows a reliable assessment of the effect of the carbides with varying strength level of the alloys and gives an opportunity to evaluate their role in different Fe-C-X alloys. The materials were again processed in a Pfeiffer VSG100 incremental vacuum melting and casting unit under an argon gas atmosphere. Subsequently, hot rolling was performed till 1.5 mm and an appropriate heat treatment was applied to obtain two main conditions: a martensitic as-quenched (as-Q) and subsequent quenched and tempered (Q&T) state, with carbides generated during tempering. A variation in the tempering time was further applied to change the carbide characteristics in the Q&T matrix allowing to thoroughly analyze the trapping ability of the carbides. Tensile specimens without a notch were prepared for these alloys with the tensile axis parallel to the rolling direction. The carbide characteristics were also studied in a ferritic microstructure since this facilitates the microstructural characterization. NbC and TiC were selected for this purpose and the experimental considered steels contained 0.013 wt % C–0.1% Nb and 0.025 wt % C–0.09 % Ti.

#### 2.1.3. Microstructural Characterization

Vickers hardness measurements were performed to determine the hardness level and the tempering temperature at which secondary hardening was most effective for the Fe-C-X alloys. Hardness measurements were performed with a drop weight of 2 kg and a pyramidal diamond indenter tip. The microstructures were investigated by light optical microscopy (LOM) and scanning electron microscopy (SEM) (FEI Quanta FEG 450, ThermoFisher Scientific, Hillsboro, OR, USA), transmission electron microscopy (TEM) (JEOL JEM-2200FS, JEOL, Tokyo, Japan), scanning transmission electron microscopy (STEM) analysis, as well as energy dispersive X-ray (EDX) spectroscopy allowed to characterize specific microstructural features such as carbides in terms of size, size distribution and morphology. Therefore, carbon replicas and thin foils were prepared. Carbon replication was made by sputtering carbon on top of a polished (1 μm) sample, followed by carbide extraction from the sample by putting the sample in a Nital 4% solution. Thin foil samples were prepared by grinding and polishing the samples to a thickness below 100 μm. Subsequently, the thin foils were electropolished, using a TenuPol-5 electropolishing unit in a 10% perchloric acid and 90% acetic acid solution. Fracture surfaces were analyzed by SEM. H assisted cracks were investigated by SEM and electron backscatter diffraction (EBSD) to characterize their morphology and crystallographic characteristics. The detection of blisters was done by LOM surface imaging permitting determination of their morphology, distribution, size, and areal density. Cross sections were analyzed to obtain information on the morphology and depth of internal cracks in H charged samples.

### 2.2. Determination of the Degree of Hydrogen-Induced Mechanical Degradation

By comparing tensile tests performed in air with tests done on in situ charged, H-saturated samples, the impact of H on the mechanical properties was determined. H was introduced in the alloys by electrochemical pre-charging using a 1 g/L thiourea 0.5 M H_2_SO_4_ solution at a current density of 0.8 mA/cm^2^ for 1 or 2 h, depending on the H saturation time. In situ charging continued during the tensile test. The conditions were chosen in such a way that they did not create blisters or any internal damage [51], which has been demonstrated to be important in earlier work [47]. The tensile tests were done at a cross-head displacement speed of 5 or 0.05 mm/min, which corresponds with a strain rate of 1.11 × 10^−3^ or 0.0111 × 10^−3^ s^−1^, respectively. The *%HE* is calculated to compare the sensitivity to H-induced mechanical degradation and defined as [45]:

(1) $$\%HE=100\;\left(1-\;\frac{\varepsilon_{ch}}{\varepsilon_{un}}\right)$$

with *ε_ch_* and *ε_un_* being the elongation of the charged and uncharged sample, respectively. Hence, the %HE varies between 0 and 1, with 0 meaning that there is no ductility loss and the material is insensitive to *HE*. When an index of 1 is obtained, the ductility drop is 100% and *HE* is maximal. HAC was investigated by performing similar tensile tests. However, instead of performing the tensile test until fracture, tests were interrupted when reaching the tensile strength. As such, H assisted cracks could be investigated more efficiently. Blisters were obtained by electrochemical H charging in a 1 g/L thiourea 0.5 M H_2_SO_4_ solution with varying charging conditions, i.e., charging time and current density. No external load is applied during these tests.

### 2.3. Determination of Diffusible and Total Hydrogen Content

Melt/hot extraction was used to determine the H saturation level, for which the samples were charged electrochemically as described above. During melt extraction, the sample is kept at 1600 °C in a pulse furnace which allows measurement of the total amount of H present in the sample. The amount of diffusible hydrogen, defined as proposed by Akiyama et al. [52,53] is measured by holding the sample at 300 °C in an infrared furnace. The metallic sample releases its H as gaseous H_2_ which is dragged along by a nitrogen flow. This mixture (N_2_–H_2_) is directed to a thermal conductivity measuring cell. Essentially, the thermal conductivity of the mixture depends on the H_2_ concentration since the conductivity of H_2_ and N_2_ differs significantly. Hence, the software can calculate the H concentration based on the variation in thermal conductivity. An average of 10 samples was taken to determine both hydrogen contents.

### 2.4. Determination of Hydrogen Trapping Capacity

The H trapping sites and their activation energy were determined by performing thermal desorption spectroscopy analysis, for which circular discs with a diameter of 20 mm and a thickness of 1 mm were used. The samples were H charged, similarly as described above, and three different heating rates of 200, 600 and 1200 °C/h were used for TDS analysis. However, the applied experimental procedure required one hour between the end of H charging and the start of the TDS measurement as an appropriate vacuum level needs to be created in the analysis chamber before the TDS measurement could start. The method based on the work of Lee et al. [54,55,56] was used to determine the *E_a_* of H traps related to the peaks observed in the TDS spectra. Equation (2) is a simplification of the original formula of Kissinger [57]. Hydrogen re-trapping and diffusion is however not considered in this simplified equation.

(2) $$\frac{d\left(ln\frac{\Phi }{T_{max}^{2}}\right)}{d\left(\frac{1}{T_{max}}\right)}=\;-\;\frac{E_{a}}{R}$$

where *Φ* is the heating rate (K/min), *T_max_* (K) the TDS peak temperature, *E_a_* (J/mol) the detrapping activation energy for specific H trapping sites associated with *T_max_* and *R* (J·K^−1^·mol^−1^) the universal gas constant. After TDS measurements using different heating rates, deconvolution of the results and determining the corresponding peak temperatures for the different traps, plotting ln(*Φ*/*T_max_*^2^) vs. (1/*T_max_*) allows to obtain the corresponding *E_a_* of the specific trapping sites.

### 2.5. Determination of Hydrogen Diffusion Coefficient

The H diffusion coefficient is determined by electrochemical permeation tests using a set-up based on the Devanathan and Stachurski method [58]. Two compartments were filled with a 0.1 M NaOH solution and a polished circular sample of 20 mm diameter and 1 mm thickness was clamped in between. Temperature was maintained constant at 25 °C and the oxygen content was limited by stirring the electrolyte using nitrogen gas bubbles. The entry side acted as the cathode by applying a current density of 3 mA/cm^2^, while the exit side (anode) was potentiostatically kept at −500 mV according to a Hg/Hg_2_SO_4_ reference electrode. The apparent H diffusion coefficient was calculated from the permeation transient using the following formula [59]:

(3) $$D_{app}\;=\frac{L^{2}}{7.7\;t}\;\left(m^{2}/\;s\right)$$

where t is the time (*s*) when the normalized steady state value has reached 0.1 and L is the sample thickness (*m*). The determination of the apparent diffusion coefficient includes the assumption of one dimensional diffusion and both initial and exit site hydrogen concentration is assumed to be zero. Although palladium coating is well known to avoid corrosion and to enhance H oxidation at the exit side, defects and/or oxides at the metal/Pd interface are easily introduced during the plating process. These heterogeneities will hence affect the H diffusion in an uncontrolled manner, which is not desirable. Since identical experimental conditions were used, the relative variations in the permeation transients are reliable. Hence, the permeation tests were done without Pd coating.

## 3. The Interaction of Hydrogen with Advanced High-Strength Steels

**Figure 1:**
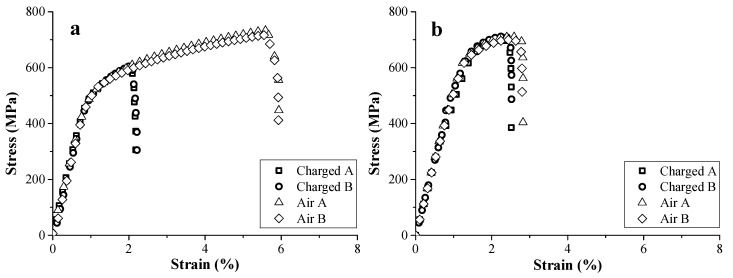
Stress-strain curves for TRIP (**a**) and HSLA (**b**). Similar tests indicated with A and B.

The impact of H on the mechanical properties of TRIP and HSLA steels was evaluated by tensile tests with in situ H charging at a deformation rate of 5 mm/min on notched tensile specimens [45]. The corresponding results on both H charged and uncharged samples are presented in Figure 1. The curves of the samples measured under the same conditions were nearly identical, confirming the good reproducibility of the test. A high HE degree of 60% was obtained for TRIP steel, while only 8% of ductility loss was found for HSLA steel. This was correlated to the presence of Nb- and Ti-based precipitates, providing effective trapping sites for H as such decreasing the H diffusivity. Generally, carbide addition has been stated to improve the HE resistance [38,39,60]. The obtained degree of HE, based on the elongation, was in good agreement with the one based on the reduction of area determined by the fracture surfaces [45]. Moreover, the fracture surface of the uncharged samples showed a considerable ductile necking zone, while the H charged samples showed a brittle transgranular cleavage failure near the edges with transition zone to some ductile features in the center of the specimen.

The tensile specimens contained a notch to control fracture initiation. However, the presence of this notch induces a specific stress distribution in the sample. To evaluate whether this would influence the failure mechanism in the presence of H, notched tensile samples were compared with unnotched samples for both TRIP steel and pure iron was used as a reference [61]. The HAC behavior of both materials was also compared to assess the effect of the microstructure on the general HAC behavior of a material. The materials exhibited a slightly different HAC behavior, since H assisted secondary crack formation occurred to a much lower extent in pure iron than in TRIP steel. HAC analysis showed that crack initiation is more stress-controlled for TRIP steel, while being strain-controlled for pure iron as a much smaller number of secondary cracks was present in pure iron which were generally wider compared to those in TRIP steel. More deformation was present on the surface of the pure iron samples as well. Both effect result from the ductile nature of pure iron, allowing more crack blunting and sample deformation prior to fracture. Sofronis and McMeeking [62] showed that the hydrostatic stress effect on the H distribution at a notch or crack is more significant for high-strength steels. Softer materials will accommodate the arising stresses more easily during plastic deformation. Therefore, a larger amount of lattice defects will be present in these regions which will trap H and hence impede H diffusion to critical high stress regions. Therefore, a strain-induced crack propagation of the principal crack is promoted, rather than stress-induced as observed for TRIP steel.

**Figure 2:**
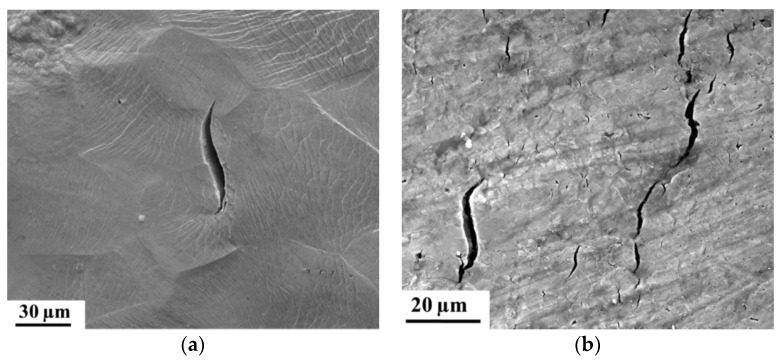
SEM images showing similar S-shape of crack for pure iron (**a**) and TRIP steel (**b**).

Further crack propagation of the secondary cracks was for both materials mainly stress-controlled. Hence, H related cracks exhibited a similar S-shape, independently of the microstructure type (cf. Figure 2). The observations in [61] indicate that the cracks propagate in two stages. First, an initiated crack propagates perpendicular to the tensile direction, which is a direct result of mode I crack tip opening in a uniaxial load situation. At a certain point (stage two), the crack starts interacting with the stress field of the approaching principal crack, which started at the notch root, causing a deviation of its crack tip by shearing in a direction determined by the local stress field. Nevertheless, the kinetics of crack initiation and propagation were presumably affected by the present microstructure and will hence occur faster for TRIP steel. Therefore, a higher number of H related cracks were formed in the TRIP steel allowing easier crack propagation. The presence of a notch similarly accelerates the HAC phenomenon, without affecting other HAC characteristics. Therefore, independently of the material’s microstructure or the presence of a notch, H assisted cracks showed a characteristic S-shape and stepwise coalescence occurred. Therefore, it was concluded that the stress state surrounding the crack tip has a major impact on the HAC features, while the stress concentration induced by the notch had little effect.

**Figure 3:**
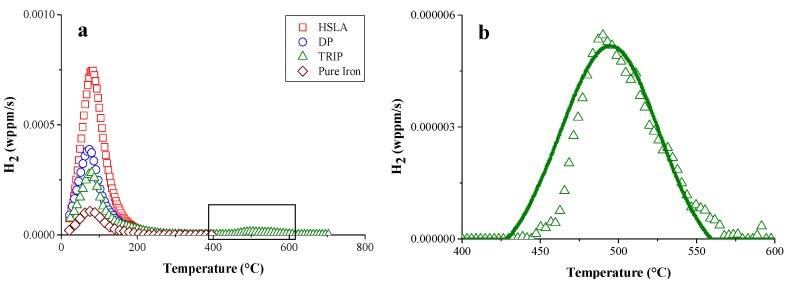
TDS spectra of HSLA, DP, TRIP steel and pure iron (**a**) with enlarged the high temperature peak of the TRIP (green) steel (**b**) (heating rate: 400 °C/h).

The same HSS as well as DP and again pure iron, as a reference, were studied further by TDS and hot extraction experiments to evaluate the H interaction with their specific microstructural constituents [44]. The corresponding TDS spectra are presented in Figure 3a. The HSLA steel showed the highest TDS peak, while it contained less diffusible H, determined by hot extraction, compared to TRIP for instance. This was again correlated to the presence of Ti- and Nb-based precipitates, lowering the H diffusivity and thus increasing the HE resistance of HSLA (cf. Figure 1). Furthermore, TRIP showed a high temperature peak, which was not observed for the other materials. This peak is enlarged in Figure 3b. To correlate it to a microstructural feature, further analysis was performed.

**Figure 4:**
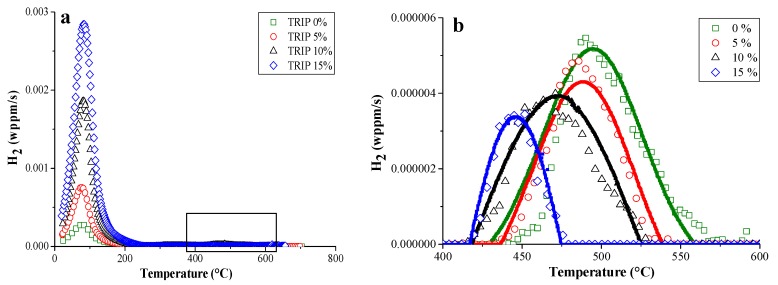
Effect of cold deformation on the TDS spectrum of the TRIP steel (**a**); with enlarged the high temperature peaks (**b**) (heating rate: 400 °C/h).

**Figure 5:**
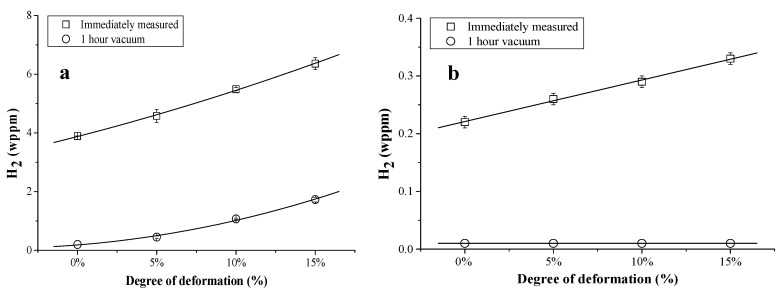
Hot extraction results immediately after H charging and after 1 h under vacuum as a function of the degree of cold deformation for TRIP (**a**) and pure iron (**b**).

TDS was also done on TRIP steel and pure iron (as a reference material), in both undeformed and cold deformed (0–5–10–15%) conditions [42]. The corresponding TDS spectra are presented in Figure 4. The TDS spectra of TRIP consist of a single low temperature peak with significantly increasing intensity with increasing deformation and a small high temperature peak, which was also affected by the amount of cold deformation. Additional characterization techniques, such as X-ray diffraction, differential scanning calorimetry and scanning electron microscopy, were used to correlate the different peaks with the microstructural evolution induced by deformation. The high temperature peak was associated with the presence of retained austenite in TRIP steel. Indeed, when the deformation % in TRIP samples increases, the amount of retained austenite decreases since it transforms into martensite which explains the decreasing peak height with increasing amount of deformation. It was demonstrated [42] that this H was introduced in the material during processing. A comparison with pure iron was done to evaluate the low temperature peak. Based on the hot extraction results (cf. Figure 5), the increase of cold deformation in pure iron, i.e., the increase in dislocation density in the material, was detectable immediately after H charging, although no difference in H content was observed after 1 hour of vacuum, which is the experimental requirement to be able to start the TDS analysis. Hence, it could be concluded that H trapped at dislocations is not detectable by TDS when operating under those specific requirements. Moreover, a comparison with pure iron and the diffusivity measurements allowed us to correlate the low temperature peak mainly with martensite formation formed by deformation-induced transformation in TRIP steel.

**Figure 6:**
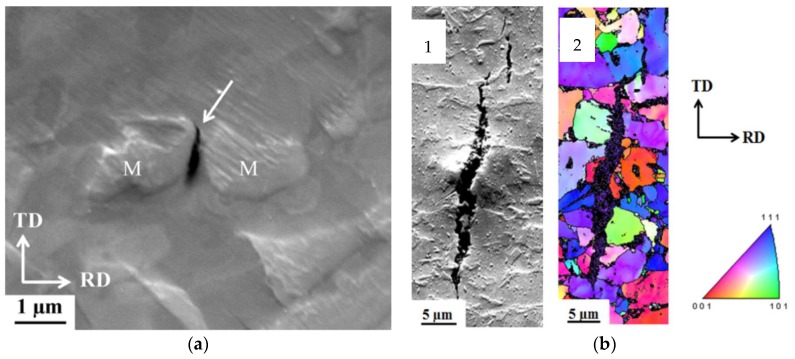
SEM image of initiating crack in TRIP between two martensitic (M) regions (**a**). SEM image of propagating crack (**b-1**) and [001] IPF map (**b-2**).

In another work, the HAC initiation and propagation was extensively investigated for TRIP steel [63,64]. 85% of crack initiations occurred in or along martensitic regions (cf. Figure 6). The other 15% of the initiating cracks were in the ferrite and bainite grain centers. No cracks were observed along non-martensitic grain boundaries. More H is present around and in the martensitic islands in comparison to neighboring ferrite or bainite. This is linked to (i) freshly formed transformation-induced martensite which is supersaturated with H, since it originates from H containing retained austenite, which has a higher solubility (cf. Figure 4); (ii) interfaces in martensite and (iii) dislocations surrounding the martensite, which are formed due to the volume expansion during phase transformation, acting as H traps. Moreover, the high HE susceptibility of martensite makes this phase very prone to HAC after application of stress when it is H-enriched. The dominating mechanism for crack initiation was found to be martensite-martensite interface decohesion, implying a H Enhanced Interface Decohesion (HEIDE) mechanism, which is a variant of the often cited HEDE mechanism [24,65,66]. In HEIDE, H enhances specifically the decohesion across the martensitic interface. Furthermore, the crack propagation mode was found to be both inter- and transgranular, although the latter was the main one (cf. Figure 6). Although the crack propagates mainly transgranularly, it is not a cleavage fracture given that the orientation of the crack does not change when going from one grain to another. No relation was found between grain orientation and crack initiation and nucleation.

**Figure 7:**
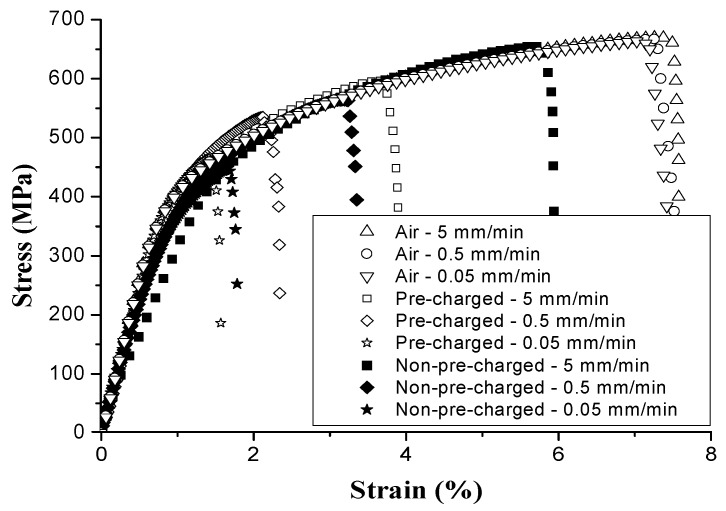
Stress-strain curves of H pre- and non-pre-charged DP at varying deformation speeds.

The H-induced mechanical degradation of DP was studied by tensile tests under variable H charging conditions, as presented in Figure 7 [43]. The ductility loss increased with H charging time until a maximal embrittlement of 50% was found, i.e., for a H-saturated sample, after which additional charging did not result in more embrittlement [43]. The influence of the cross-head deformation speed on H-saturated samples allowed evaluating the effect of H diffusion during the test. HE increased when lowering the test speed, as H was enabled to diffuse to critical regions ahead of the crack tip during the test.

**Figure 8:**
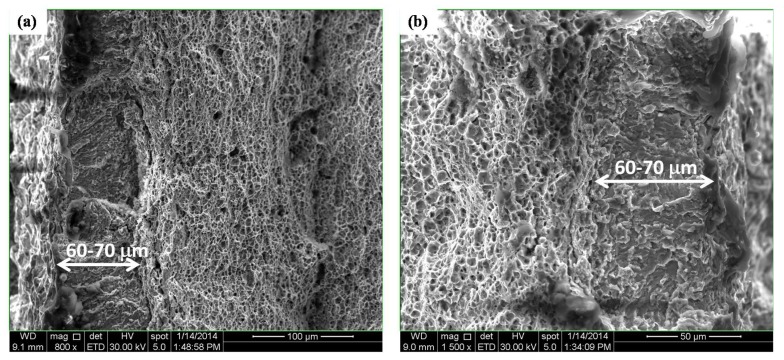
Detailed SEM image of both left (**a**) and right (**b**) side of the fracture surface of the sample tested at cross-head deformation speed of 5 mm/min without H pre-charging. The brittle zone is shown.

Different cross-head deformation speeds were also applied on uncharged samples. Comparison between the outcomes of the charged and uncharged tests allowed to visualize the effective H diffusion distance into the sample during the test. The H distance *x* (cm) can be calculated by taking the square root of the product of the diffusion coefficient *D* (cm^2^/s) and the test time *t* (s), i.e., *x* = (*D* x *t*)^1/2^ [33]. The distances H can diffuse at 5, 0.5 and 0.05 mm/min were about 65, 168 and 410 μm, respectively, which confirms the increased importance of H diffusion at lower deformation speeds. A fractography study indeed proved that the brittle H-induced features were present over a distance equivalent to the distance H can diffuse during the test (cf. Figure 8). Tests performed at the lowest cross-head speed, revealed brittle cleavage appearances in the central region which was correlated to the MnS segregation line that is prone to H-induced fracture.

**Figure 9:**
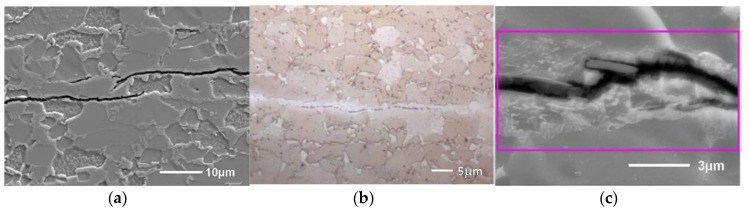
SEM (**a**) and LOM (**b**) images of DP revealing HIC together with SEM image (**c**) indicating the MnS containing segregation line in the center of the sample.

**Figure 10:**
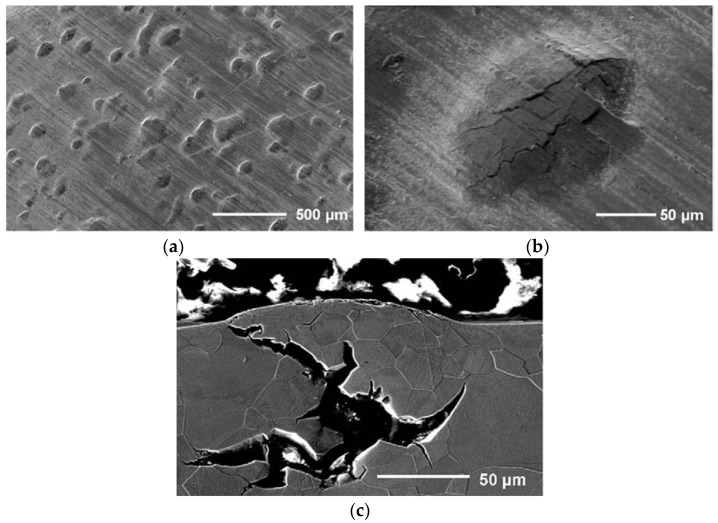
SEM images of surface (**a**, **b**) and cross section (**c**) of a crack propagation in pure iron charged for 1 h at 50 mA/cm^2^ in arsenic-poisoned electrolyte.

These HSS and pure iron, as a reference, were also charged with H without external load application using various severe charging conditions to evaluate both surface damage (H-induced blisters) and internal damage (H-induced cracks). To do so, different electrolytes, charging current densities and charging times were used [67]. Internal damage in DP samples was observed by the presence of cracks situated in the middle of the sample and propagating along the rolling direction (cf. Figure 9). HSLA did not show cracks for the same charging conditions. Cracks were identified to be both inter- and intragranular. Mn segregations were demonstrated for the DP and TRIP steel and elongated MnS inclusions were even clearly identified in the middle of the crack for the DP steel. This suggested that these second phase particles might play a role in the initiation of H-induced cracks. Similar results were obtained in [68,69], where inclusions which were hard, brittle, and incoherent with the matrix, such as manganese sulfides were recognized to be harmful to H-induced cracking. Similar blister formation and H-induced crack analysis was performed on pure iron as well. Pure iron showed faster blister formation compared to the HSS and was found to be more susceptible to H surface damage due to its softer matrix, as shown in Figure 10.

## 4. The Interaction of Hydrogen with Lab-Cast Fe-C Alloys

**Figure 11:**
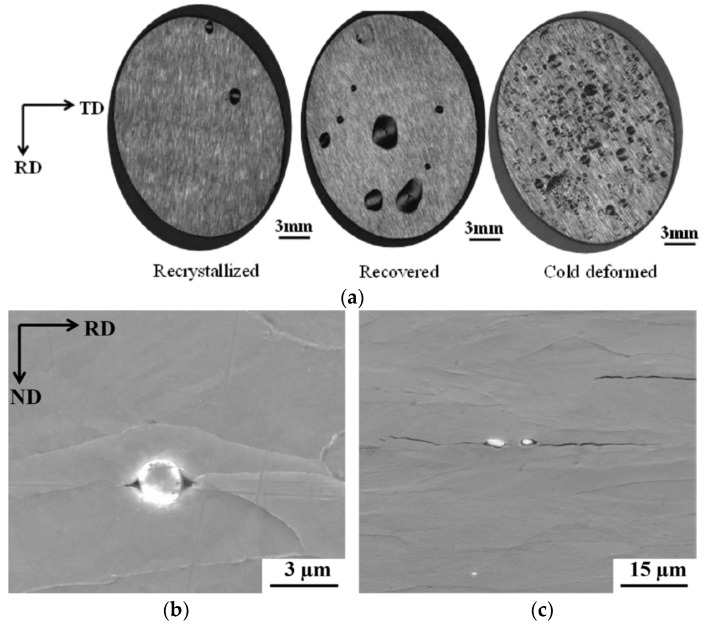
Blister surface appearance (**a**) of recrystallized, recovered, and cold deformed ULC steel charged at 5 mA/cm^2^ for 2 days together with SEM images revealing crack initiation at alumina particle (**b**, **c**).

The H/material interaction was further studied in generic alloys to avoid the effect of complex microstructural characteristics. A H-induced crack analysis was also done in an ultra-low carbon steel by exposing it to electrochemical H charging [49]. Three material conditions, i.e., cold deformed, recovered, and recrystallized, were considered to assess the impact of deformation-induced defects, such as dislocations and microvoids [70], on the HIC sensitivity. Generally, H diffusion is hindered by such defects, which stimulates H accumulation and thus causes easier crack initiation at these sites. Therefore, the H-induced blister formation is promoted by these defects, as shown in Figure 11. However, a certain critical concentration of H needs to be reached before initiation of blisters occur. If this critical concentration is reached, H recombination will occur, and blister formation takes place according to the internal pressure theory [66], which was confirmed by EBSD in [49]. The H charging time at a specific charging current density required to achieve this concentration is decreased for deformed material compared to heat treated material since a higher amount of crack initiation sites are present in deformed material due to the presence of a higher density of deformation-induced defects. Initiation of HICs in recrystallized material was related to debonding of inclusions which were found in the material, as illustrated in Figure 11. The same observations were made by Tiegel et al. [71]. They stated that H accumulation occurred at the interfaces of incoherent particles, causing an interface failure. Moreover, the high H concentration created vacancy stabilization providing the required space to form molecular H and the consequential internal pressure build-up resulted into cracks. Additionally, the cracking behavior was also largely affected by the applied current density (10 vs. 20 mA/cm^2^), which was linked to a change in the internal crack morphology. Cracks were dominantly transgranular at low current density. The favorable crack propagation paths were associated to high dislocation density slip planes. While intergranular crack propagation along high angle grain boundaries occurred at higher current density.

**Figure 12:**
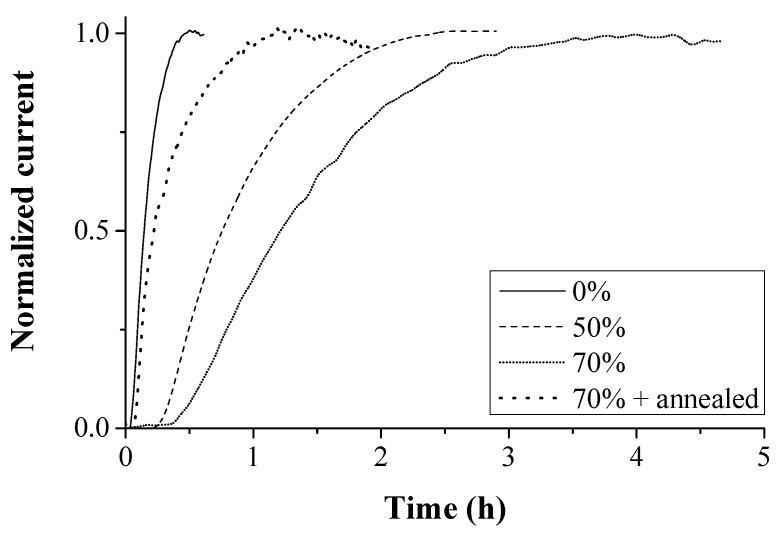
Permeation transients of Armco iron in specific cold deformed conditions.

The influence of deformation-induced defects was studied further by permeation experiments. Since inclusions were present in the ULC steel, Armco pure iron was used for this purpose [50]. The material was reduced in thickness by 50 and 70% of cold rolling. Figure 12 shows how the permeation transient is affected by the microstructural changes, i.e., a H diffusivity decrease was linked to the presence of deformation-induced defects, such as dislocations and microvoids [70]. When the material was annealed to release the stresses of the 70% cold rolled grade, a higher diffusivity was observed, implying less H traps present in the material. This was more linked to rearrangements of dislocations and internal stress relief than to a considerable decrease of dislocation density [72].

The effect of H charging on the mechanical properties of Fe-C alloys was studied in [46,47,48]. Different phases, such as pearlite, bainite and martensite were generated in a 0.2% C Fe-C alloy by an appropriate heat treatment, referred to as P2, B2 and M2, respectively. An increase in the carbon content up to 0.4% was established for the bainitic grade, i.e., B4. Tensile tests were done at 5 mm/min on notched specimens and a significantly different HE degree was observed. The obtained degrees of HE were correlated with the amount of H present in the materials (cf. Table 4). A distinction between the amount of diffusible H and the total H was made, since diffusible H has been argued to play a crucial role in H-induced failure [5,73,74]. For the 0.2% C Fe-C alloys, the pearlitic microstructure was more susceptible to HE compared to the bainitic and martensitic grade, which exhibited a similar HE response. This was linked to the higher diffusible H content and H diffusivity for P2. Increasing the C% to 0.4% for the bainitic microstructure resulted in an increased HE due to the larger amount of diffusible H. The yield strength was also affected by H charging due to solid solution strengthening caused by the interstitial H as the increase in yield strength was correlated with the total amount of H determined by melt extraction, as observed as well in [5]. Tensile tests performed at a lower speed of 0.05 mm/min allowed to further elaborate the synergetic effect of both the amount of diffusible H and the H diffusion distance during the test. An additional ductility loss was observed at a slower cross-head speed, which was the lowest for the martensitic grade (22→30%), as this material showed the lowest H diffusivity. Although adding carbon resulted in a higher amount of diffusible H, a lower HE increase was obtained for B4 (40→63%) compared to B2 (21→50%) when lowering the cross-head deformation speed. This was related to the lower H diffusion coefficient of B4 (3.40 × 10^−10^ m^2^/s) compared to B2 (6.71 × 10^−10^ m^2^/s), as demonstrated in [48].

## 5. The Interaction of Hydrogen with Lab-Cast Fe-C-X Alloys

**Figure 13:**
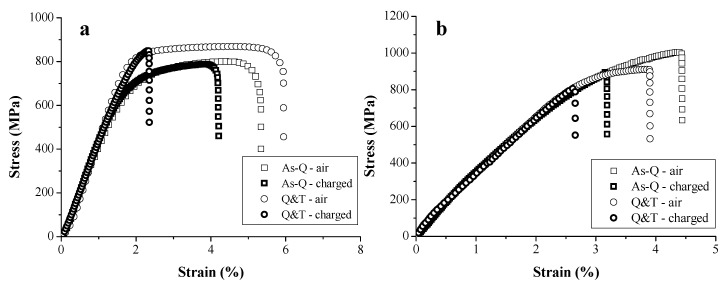
Stress-strain curves for (**a**) Fe-C-Ti; (**b**) and Fe-C-V alloy B at a cross-head deformation speed of 5 mm/min of uncharged (air) and H-saturated (charged) samples.

The effect of carbide addition was evaluated in several types of Fe-C-X alloys [51,75,76,77,78,79], with X = Ti, V, Mo, Cr and W, respectively. At first, a martensitic microstructure was considered where the as-Q and Q&T condition, containing tempered induced carbides, were compared. Tensile tests were done in air and on H-saturated unnotched specimen at 5 mm/min. The stress-strain curves for the Fe-C-Ti and Fe-C-V alloy B (medium C content) in both conditions are depicted in Figure 13. Tempering caused an increase in strength level and ductility for Fe-C-Ti whereas the opposite effect was observed for Fe-C-V. Only a limited amount of plastic deformation was present for the Fe-C-V alloy since it includes a fast dissolution of carbides during austenitizing, resulting in a dense C rich martensitic microstructure and hence a more brittle material after quenching. For the Q&T condition, the secondary hardening effect was pronounced for Fe-C-Ti, while a decrease in dislocation density overtook the strengthening effect of the precipitates for Fe-C-V. The HE degree increased upon tempering when comparing the as-Q with the Q&T condition for Fe-C-Ti (21→60%) and Fe-C-V (28→32%).

**Figure 14:**
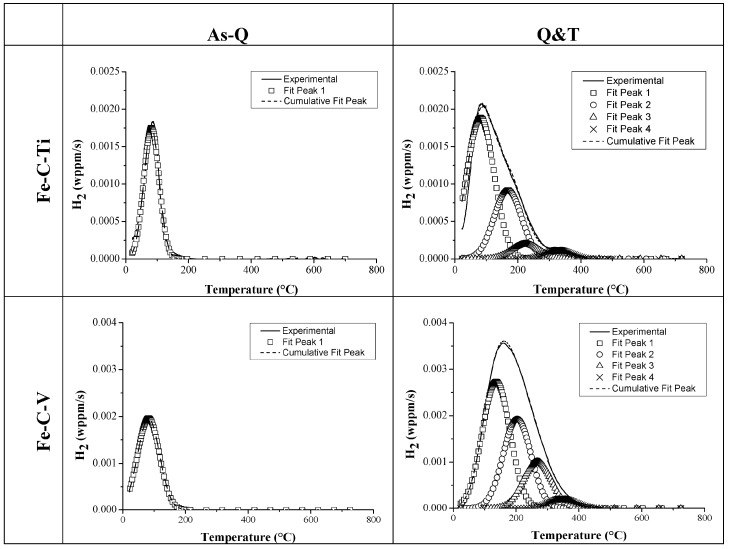
TDS curves of Fe-C-X grades (alloy B) in the as-Q and Q&T condition (heating rate: 600 °C/h).

The H/material interaction was studied further to comprehend the underlying reasoning of the obtained HE%. TDS was done on all Fe-C-X materials for both the as-Q and Q&T condition. The TDS spectra of Fe-C-Ti and Fe-C-V alloy B in both conditions, together with their deconvoluted peaks, are presented in Figure 14. For the as-Q condition, only one peak was observed showing activation energies of about 27–30 kJ/mol. This first peak was therefore attributed to H trapped by martensitic lath boundaries [80]. Although H trapped at dislocations shows an E_a_ in the same range [9,70,81,82], this trapping site is assumed to be largely undetectable due to the experimental requirements to perform the TDS analysis (cf. Figure 4 and Figure 5) [42,83]. Although incoherent large TiC particles were present in the as-Q condition of Fe-C-Ti [51], a single peak was present indicating that no H was trapped from electrochemical charging at their interface. This confirmed that gaseous H charging at elevated temperature was required to charge these large particles [40] and H from the gaseous charging being presumably trapped inside the carbide [37] instead of at its interface, which will be further analyzed.

**Figure 15:**
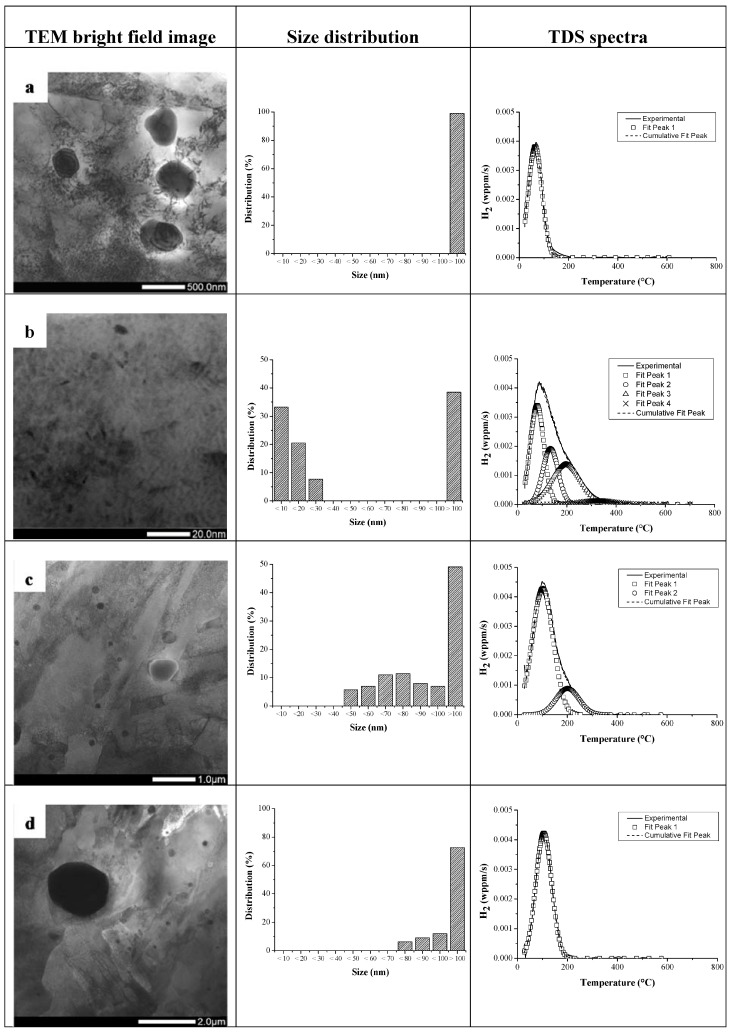
Representative TEM bright field image, carbides size distribution and TDS spectrum for alloy C in the as-Q (**a**); Q&T 1 h (**b**); Q&T 10 h (**c**) and Q&T 20 h (**d**) condition (heating rate: 600 °C/h).

When considering the Q&T condition, a significantly different trapping behavior was revealed. The additional peaks in the spectra were attributed to the presence of small TiC and V_4_C_3_ carbides which can trap a high amount of H, resulting in three peaks with activation energies in the range of 50–71 kJ/mol. The first deconvoluted peak was slightly increased for the Q&T condition compared to the as-Q condition, which was attributed to the slower H diffusivity due to the presence of the precipitates, as determined by permeation [51,75,76,77,78]. Since the trapping ability was found to be dependent on the carbide size, the tempering time was increased (10 and 20 h) to verify the size dependent trapping ability of TiC, as illustrated in Figure 15. Representative TEM bright field images are shown for each condition, together with the corresponding carbide size distribution map to evaluate the increase in carbide size with tempering time. The related TDS spectra is given as well to correlate the deconvoluted peaks to the microstructural characteristics. The carbide related TDS peaks decreased with tempering time, which was linked to the decreased total interfacial area between the carbides and the matrix. As such, it was confirmed that H was trapped at the carbide/matrix interface. Moreover, it was shown that no H was trapped by TiC larger than 70 nm by tempering the material for 20 h, as shown in Figure 15d. The peak shift obtained when comparing Figure 15a with Figure 15d is linked to the increased elastic strain fields surrounding the large incoherent TiC [51].

**Figure 16:**
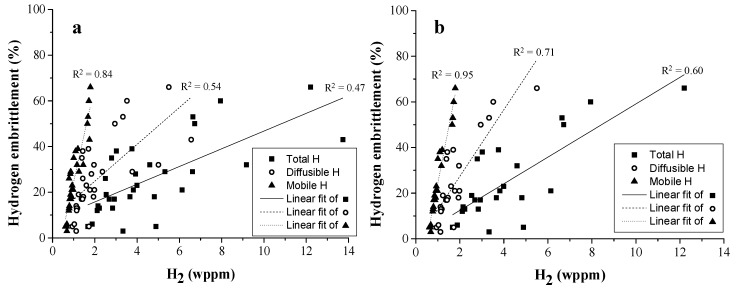
Degree of HE vs. the total, diffusible and mobile H content for all alloys of the Fe-C-X grades in the as-Q and Q&T condition (**a**); The Fe-C-V alloys were excluded in (**b**) to illustrate the correlation between mobile H and HE for alloys which failed past the macroscopic yield strength.

Three different types of H were distinguished in the detailed study on Fe-C-X alloys, i.e., the total H amount measured by melt extraction, the diffusible H content measured by hot extraction and the mobile H content, which was defined as the amount of H which was released before the TDS measurement due to the abovementioned experimental requirements to achieve a vacuum level in the analysis chamber. This mobile H amount was calculated by subtracting the amount determined by TDS from the diffusible H content. This mobile H is correlated to H trapped at dislocations which is undetectable by that specific TDS set-up [83]. To analyze the correlation between the HE degree and the type and amount of H, a linear fitting of the different types of H (i.e., total, diffusible, and mobile) and HE% is made (cf. Figure 16a). For this purpose, all alloys for each Fe-C-X grade (cf. Table 3) in the as-Q and Q&T condition were included (30 different materials conditions). The correlation improved for total over diffusible to mobile H. Moreover, when the Fe-C-V materials were excluded, which is a valid assumption since they did not show any significant plastic deformation, (cf. Figure 16b), a R^2^ of 95% between the degree of HE and the amount of mobile H was achieved. This clearly indicated the crucial role of H trapped by dislocations on the HE during a uniaxial tensile test which is linked to the enhancement of the dislocation mobility by the presence of H as proposed by the HELP mechanism [83]. Therefore, these experimental results present a very nice confirmation of the HELP theory.

**Figure 17:**
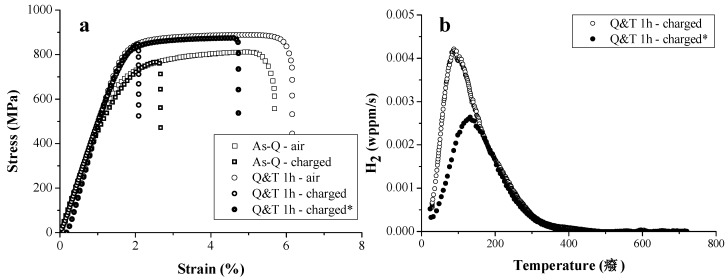
Stress-strain curves (**a**) and corresponding TDS spectra (**b**) for Fe-C-Ti alloy C in the as-Q and Q&T 1h condition. H charging was applied till saturation (charged) and until a similar H amount as as-Q was obtained for Q&T 1 h-charged*.

One remarkable result was the increased HE susceptibility for Q&T samples despite the presence of H trapping carbides (cf. Figure 13 and Figure 14). Evidently, the question arose whether the presence of these precipitates is a good strategy to enhance the resistance to HE. Since a clear correlation between the amount of H and the HE degree was found (cf. Figure 16), an alternative charging procedure was applied. So far, the materials were charged electrochemically till H saturation, which implies that the amount of H present in the as-Q and Q&T condition upon testing was not equal. Indeed, for example a H-saturated Q&T material having TiC contained about double the amount of H than its as-Q variant. Consequently, a similar H content compared to the as-Q state was charged in Q&T condition of Fe-C-Ti in a second series of tensile tests. This condition will be referred to as Q&T ‘charged*’. These materials were compared with the H-saturated as-Q and Q&T samples and the corresponding stress-strain curves are displayed in Figure 17a. The HE resistance increased considerably when the tempered condition was compared to the as-Q state when a similar amount of H was present. Therefore, an appropriate next step is to evaluate the H distribution in the non-saturated Q&T samples (charged*) over the different traps by TDS and to compare the resulting spectrum with the TDS spectrum of the Q&T sample which was H-saturated (charged), as shown in Figure 17b. This comparison indicated that the trapping sites with the higher activation energy, i.e., those linked with TiC carbides, were first filled. The first peak, mainly associated with H at lath boundaries, had not yet been filled in the non-saturated materials. Therefore, H was first trapped by the deeper trapping sites, which have the higher E_a_. Consequently, a higher proportion of H is strongly trapped and hence the non-saturated samples have a relatively lower amount of mobile H which improves the HE resistance. This demonstrates that TiC are indeed beneficial to reduce the susceptibility to HE. Hence, the beneficial effect of carbide addition depends on the carbides characteristics, the charging conditions and effective amount of hydrogen induced in the material.

**Figure 18:**
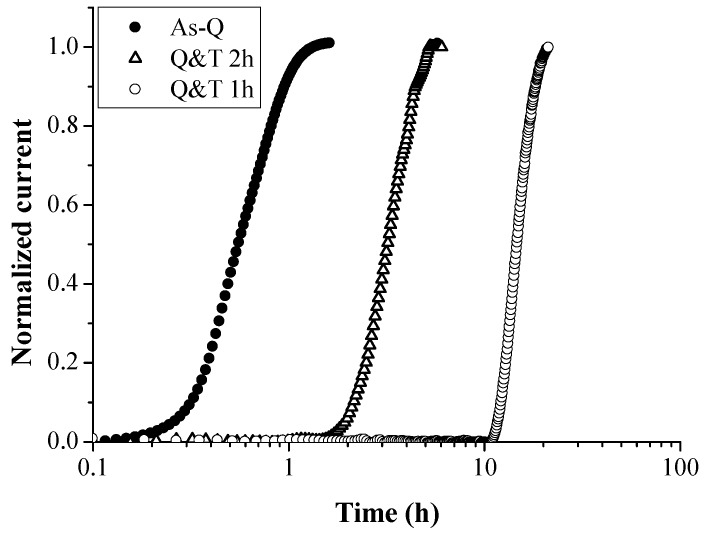
Permeation curves for Fe-C-Ti alloy C in the as-Q, Q&T 1 h and Q&T 2 h condition.

An alternative way to evaluate the available trapping sites was done by performing permeation tests, as presented in Figure 18. The H diffusivity increased from Q&T 1 h < Q&T 2 h < as-Q which was in the opposite order as the amount of available strong trapping sites. When tempering was done for 2 h, the TDS peaks linked to H at TiC, i.e., peak 2, 3 and 4 in Figure 15b, considerably decreased. Since the amount of interface between carbides and matrix decreased when the precipitates grew, and their interface became more incoherent, the present results indicated that H was trapped at this interface rather than inside the carbides. These observations are in good agreement with those obtained by Wei and Tsuzaki [40,41], Pérez Escobar et al. [84] and density functional theory calculations by Di Stefano [37].

**Figure 19:**
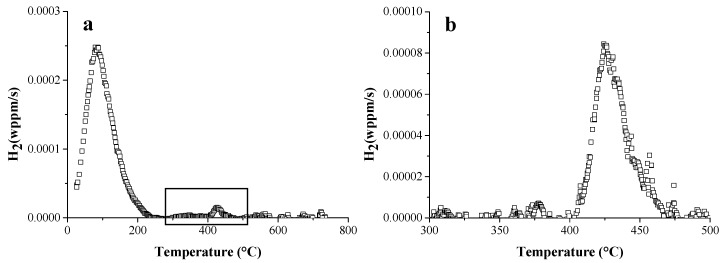
TDS spectrum of NbC containing ferritic steel after both electrochemical and gaseous charging (**a**); The high temperature peak linked with H inside NbC is presented in (**b**) (heating rate: 400 °C/h).

The H interaction with carbides was also considered in a ferritic microstructure for several carbide-forming elements. At first, the H trapping ability of NbC was considered by TDS in an experimental steel containing 0.013 wt % C–0.1 % Nb [85]. The sample was, next to gaseously charged at elevated temperature and atmospheric pressure, also electrochemically charged before the measurement. The details on the gaseous hydrogen charging procedure can be found elsewhere [85]. This resulted in a curve showing two distinct peaks, a low temperature peak and a high temperature peak (cf. Figure 19). The low temperature peak was attributed to the H that was introduced by electrochemical charging and trapped at grain boundaries, with activation energies ranging from 24–33 kJ/mol and at the interface of the smaller precipitates, with *E_a_* of about 23–48 kJ/mol. The high temperature peak was attributed to the H that is introduced by gaseous charging and is trapped inside the vacancies of the incoherent NbC precipitates, with an activation energy ranging between 63 and 68 kJ/mol. Gaseous charging is necessary to provide the required energy to trap the H in the vacancies of the precipitates.

**Figure 20:**
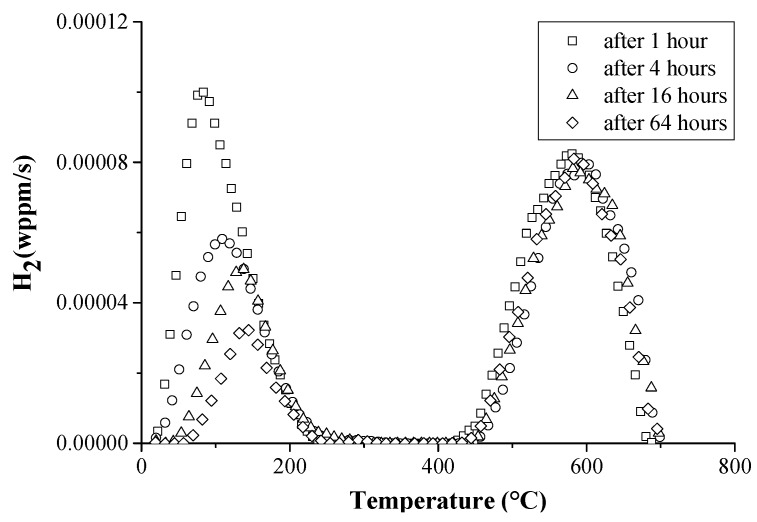
TDS spectra of TiC containing ferritic steel annealed at 800 °C in gaseous H_2_ atmosphere, charged electrochemically and submitted to specified desorption times in vacuum (heating rate: 400 °C/h).

The next experimental steel, with 0.025 wt % C and 0.09 wt %Ti, showed a microstructure consisting of small ferrite grains and nanometer size TiC precipitates [84]. The steel contained some H in irreversible traps, which were TiC, and which originated from the hot rolling of the steel. After annealing in H_2_, the TDS spectra contained a high temperature peak, for which both the peak area and peak temperature increased with higher annealing temperature, which was related to an increased carbide vacancy concentration at higher annealing temperature, which may act as potential H traps. Therefore, this peak was attributed to irreversible trapping by TiC particles. The TDS spectra for samples annealed in H_2_ atmosphere at 800 °C and sequentially electrochemically charged for 1 h at 0.8 mA/cm^2^ are presented in Figure 20. A low and high temperature peak was revealed. With increased desorption time, the low peak decreased in height, whereas the high temperature peak did not change significantly. Therefore, the low temperature peak was correlated with reversible traps, i.e., grain boundaries, while the high temperature peak was associated with irreversible trapping by the TiC particles. The amount of H in the reversible traps desorbed gradually with increased desorption time, whereas practically all H trapped by TiC remained in the material. The irreversibly trapped H by TiC in the high temperature peak showed an *E_a_* of about 145 kJ/mol.

## 6. Conclusions

The H/material interaction was studied in this work. In situ tensile testing was done to evaluate the HE sensitivity, whereas the H content was determined by hot and melt extraction. TDS analysis further revealed the H trapping capacity and permeation experiments allowed the determination of the H diffusivity. Blisters or HIC were induced by severe electrochemical charging and HAC was assessed by interrupting the in situ tensile tests to understand the complete failure process in terms of crack initiation and propagation. These H characterization techniques were related with a thorough microstructural analysis of the different considered materials.

The interaction of H with multiphase high-strength steels was studied and a combined influence of both the amount of H and the diffusivity of H determined the observed HE degrees. HSLA steel showed the highest resistance to HE (8% ductility loss) which was linked to the presence of Ti- and Nb-based precipitates. TDS demonstrated that the retained austenite in TRIP showed a high activation energy trap for H, present from the processing of the material. Though, since retained austenite transformed into martensite during tensile deformation, a high HE sensitivity of 60% was obtained for this material. Further SEM and EBSD analysis showed that initiation of HAC in TRIP occurred at the martensitic islands, which is established on a H-enhanced interface decohesion mechanism. Furthermore, the HAC characteristics were not affected by the presence of a notch in the tensile sample geometry. Lastly, a fractographic SEM analysis on DP steel clearly visualized the effect of H diffusion during an in situ tensile test; the calculated H diffusion distance equals the observed transition between a H-induced brittle and ductile fracture. Moreover, HIC was demonstrated to initiate at the MnS inclusions present in this DP steel.

Nevertheless, these multiphase high-strength steels contain a complex microstructure, which motivated the fundamental H/material interaction in single phase microstructures, such as ferrite, pearlite, bainite and martensite. ULC steel, pure iron and generic Fe-C alloys were used for this purpose. Initiation of HIC in recrystallized ULC steel was correlated with debonding of inclusions present in the material. Permeation experiments allowed to evaluate the influence of deformation-induced defects in pure iron. A decrease in H diffusivity was observed and linked to the presence of deformation-induced defects, such as dislocations and microvoids. The HE susceptibility was further evaluated in Fe-C alloys and both the H amount and diffusivity determined the observed degrees of HE. Finally, the H trapping ability of carbides and their effect on the susceptibility to HE was considered in generic Fe-C-X materials. TDS revealed that the carbide trapping ability was mainly determined by their size and coherency with the matrix. In addition, gaseous H charging at elevated temperature was required to charge the incoherent carbides, showing an irreversible H peak by TDS. The amount of H present in the materials, as determined by combining hot extraction, melt extraction and TDS, was correlated with the HE sensitivity. It was demonstrated that H trapped by dislocations played a determinant role in the obtained HE degrees. This was linked to an enhanced dislocation mobility in the presence of H, which provides a clear experimental support for the HELP mechanism.

## Figures and Tables

**Table 1:** Chemical compositions in wt %. Fe content in balance.

Material/Element	C	Mn	Si	Other
TRIP	0.17	1.60	0.40	1–2% Al, 0.04–0.1% P
DP	0.07	1.50	0.25	0.4–0.8% Cr + Mo
HSLA	0.07	0.95	0.00	0.08–0.12% Ti + Nb

**Table 2:** Chemical composition of the used materials in wt %. Fe content in balance.

Material/Element	C	Mn	Si	Other
“Pure Iron”	0.0015	0.0003	0.00	<0.02% Al, P
Ultra-low C	0.0214	0.2500	0.00	<0.05% Al, P, N
Armco iron	0.001	0.050	0.003	<0.005% Al, P, N
0.2% C	0.199	0.004	<0.0002	<0.0008 P, N
0.4% C	0.374	0.002	<0.0001	<0.0007 P, N

**Table 3:** Chemical compositions of the Fe-C-X materials in wt %. Fe content in balance.

Alloy Fe-C-X		C	X	Other Elements
Fe-C-Ti	Alloy A	0.099	0.380	Al: 200–300 wt. ppm Other elements: traces
	Alloy B	0.202	0.740	
	Alloy C	0.313	1.340	
Fe-C-Cr	Alloy A	0.097	1.300	
	Alloy B	0.143	1.800	
	Alloy C	0.184	2.200	
Fe-C-Mo	Alloy A	0.100	1.700	
	Alloy B	0.142	2.380	
	Alloy C	0.177	2.990	
Fe-C-W	Alloy A	0.096	2.670	
	Alloy B	0.186	6.130	
	Alloy C	0.277	8.700	
Fe-C-V	Alloy A	0.100	0.570	
	Alloy B	0.190	1.090	
	Alloy C	0.286	1.670	

**Table 4:** Melt (1600 °C) and hot (300 °C) extraction results for H-saturated samples in wppm (weighted Parts per million).

	Total H (wppm)	Diffusible H (wppm)	HE% (5 mm/min)	HE% (0.05 mm/min)
B2	8.71 ± 0.12	3.82 ± 0.06	21	50
M2	10.53 ± 0.15	4.72 ± 0.07	22	30
P2	9.69 ± 0.13	5.10 ± 0.09	52	-
B4	14.65 ± 0.16	8.73 ± 0.11	40	63
